# Reed bamboos drive skull shape evolution in bush frogs of the Western Ghats, Peninsular India

**DOI:** 10.1002/ece3.10493

**Published:** 2023-09-05

**Authors:** Kalpana Das, Mark‐Oliver Rödel, Edward Stanley, Achyuthan N. Srikanthan, Kartik Shanker, Seenapuram Palaniswamy Vijayakumar

**Affiliations:** ^1^ Museum für Naturkunde – Leibniz Institute for Evolution and Biodiversity Science Berlin Germany; ^2^ Department of Natural History, Florida Museum of Natural History University of Florida Gainesville Florida USA; ^3^ Centre for Ecological Sciences Indian Institute of Science Bangalore India; ^4^ Department of Biology Clark University Worcester Massachusetts USA

**Keywords:** 3D geometric morphometry, adaptive radiation, amphibia, anura, bush frogs, ecological opportunity, morphology

## Abstract

Reed bamboo is a major ecological and economic resource for many animals, including humans. Nonetheless, the influence of this plant's evolutionary role on the morphology of animal species remains unexplored. Here, we investigated the significance of bamboo habitats as ecological opportunities in shaping the skull morphology of bush frogs (*Raorchestes*) from the Western Ghats, Peninsular India. We applied a three‐dimensional (3D) geometric morphometric approach to capture the skull shape of 55 species of bush frogs. We visualized the skull shape variables in phylomorphospace with principal component analysis and performed phylogenetic generalized least‐squares analysis to assess the impact of cranial size (evolutionary allometry) and habitat (bamboo or non‐bamboo) on cranial shape. We quantified the morphological disparity between bamboo and non‐bamboo bush frogs' skull shape, and employed RRphylo, a phylogenetic ridge regression method, to access the evolutionary rate and rate shifts of skull shape change. The phylomorphospace delineated bamboo and non‐bamboo bush frogs. While cranial shape exhibited a significant but smaller association with size, its association with habitat type was non‐significant. We detected, however, significant differences in skull shape between the two frog groups, with bamboo frogs showing higher morphological disparity and a remarkable shift in the evolutionary rate of skull shape diversification. These findings underscore the role of reed bamboo in the evolution of skull shape in the radiation of frogs, endemic to the Western Ghats. We demonstrate that the association between the members of two distinct endemic clades (bamboo reeds and bamboo frogs) is the outcome of a deep‐time ecological opportunity that dates back to the Miocene.

## Graphical abstract

Our study highlights the key role of ecological opportunity as a driver of morphological evolution. We find that endemic bush frogs from a monophyletic group (Ochlandrae) which lives in the bamboo internodes. They have skull shapes different to non‐bamboo frogs. We uncovered a deep‐time association between bush frogs and bamboo species, dating back to the Miocene.

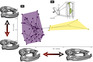

## 1 INTRODUCTION

Ecological opportunity often acts as a driver of morphological diversity (Wellborn & Langerhans, 2015). Ecological opportunity can be defined as a suite of ecological resources available to species in the absence of potential competitors (Yoder et al., 2010). Key innovations in species morphologies often pave the way to access those resources (Dumont et al., 2012; Mahler et al., 2010; Yoder et al., 2010). This can lead to morphological diversification in clades, which then acts as a precursor for adaptive radiation (Burbrink & Pyron, 2010).

Adaptive radiation in animals is often associated with changes in syntopic plants. For example, the diversity of taxa such as horses and herbivorous beetles are associated with the emergence of grasslands and angiosperms, respectively (MacFadden, 2005; Mckenna et al., 2009). Similarly, arboreality in frog clades such as the Natatanurans coincides with the recovery of forests after the K–Pg boundary (Feng et al., 2017). Among plants, bamboos exhibit intriguing associations with various animal species. Bamboos are tall grasses of the Poaceae subfamily Bambusoideae. The tribe of tropical woody bamboos (Bambuseae) occurs in the Paleo‐ as well as in the Neotropics (Wysocki et al., 2015).

Woody bamboos possess hollow culms (jointed stems), each culm segment being intersected by solid joints called nodes. The space between nodes, so‐called internodes, attracts many invertebrates and vertebrates (Arruda et al., 2016; Tu et al., 2017). This also includes frogs, which specifically use hollow, water‐filled internodes for breeding, as seen for instance in the Taiwanese *Kurixalus eiffingeri* (Lin & Kam, 2008). Similarly, bamboo forests of Madagascar harbor a great diversity of frog species using bamboo internodes as breeding sites (e.g., the microhylid genera *Platypelis* and *Anodontyhla*; Glaw & Vences, 2007). Apart from frogs, the Southeast Asian bamboo bats of the genera *Tylonycteris* and *Eudiscopus* use bamboo internodes as reproductive retreats and roost inside it (Saikia et al., 2021). Despite the well‐known importance of bamboo for various anuran species, the evolutionary impact of bamboo on the morphology of the respective frog clades has not been studied.

The Western Ghats, a 1600 km long escarpment along the west coast of Peninsular India, is home to a spectacular number of bush frogs, *Raorchestes*, a clade that has radiated in‐situ (Vijayakumar et al., 2016). Notably, certain species within this clade have developed a close association with reed bamboos, particularly from the genus *Ochlandra* (Seshadri et al., 2015; Seshadri & Bickford, 2018). These frogs exhibit a unique reproductive strategy. They enter bamboo internodes through narrow openings. Inside they deposit their direct developing eggs, and guard them against potential predators (Seshadri et al., 2015). We assume that this behavior should exert strong selection pressure on the morphology of these bush frog species, particularly on their skull shape, to facilitate the entrance through narrow holes into the internodes. This assumption is based on the data from Southeast Asian bamboo bats, roosting inside the internodes, and exhibiting morphological adaptations such as dorsoventrally flattened skulls, and padded thumbs and feet (Tu et al., 2017).

Currently, there are more than 60 known *Raorchestes* species recognized as valid. The bush frog lineages associated with bamboo belong to a monophyletic group within *Raorchestes*, the clade Ochlandrae. There are five species, *Raorchestes chalazodes*, *R. ochlandrae*, *R. manohari*, *R. uthamani*, *R. flaviocularis*, assigned to the Ochlandrae clade (Garg et al., 2021; Vijayakumar et al., 2014). Ochlandrae provides an excellent setting for understanding the significance of bamboos as an ecological opportunity in morphological diversification, suggesting the possible origin of an association between these bamboo‐dwelling bush frogs and bamboos in a common ancestor (Vijayakumar et al., 2014, 2016).

Based on the known association of reed bamboo bush frogs with bamboo habitats, coupled with their known shared ancestry, we hypothesize that reed bamboo frogs would occupy a distinct morphospace in comparison to other *Raorchestes*. Similarly, based on shared ancestry and habitat requirements, we also hypothesize, that compared to other members of the genus *Raorchestes*, the reed bamboo clade Ochlandrae shows lower disparity in skull shape. Finally, we hypothesize that reed bamboos have provided an ecological opportunity to the most recent common ancestor (MRCA) of Ochlandrae. It has been known that clades exposed to ecological opportunity tend to show shifts in morphological diversification rates (Yoder et al., 2010). Therefore, we tested whether the diversification rate in the branch originating from the most recent common ancestor (MRCA) of Ochlandrae, differs from other *Raorchestes*.

## 2 MATERIALS AND METHODS

### 2.1 Sampling

For our study, we used bush frogs collected form a previous study (Vijayakumar et al., 2014, 2016). We chose one adult, male individual from 55 available lineages/species for the micro‐CT scanning. Males were identified based on the secondary sexual characteristics such as vocal sacs and nuptial pads (Table S1). The habitat data for each lineage was available from field surveys in the Western Ghats (Vijayakumar et al., 2014). We classified each species into bamboo dwelling and non‐bamboo dwelling based on their habitat association.

### 2.2 CT scanning and processing

We performed high‐resolution micro‐computed tomography (CT) scans of entire specimens, using the X‐Radia Versa 500 X‐ray microscope (XRM; Carl Zeiss Microscopy GmbH) at the Advanced Facility for Microscopy and Microanalysis, Indian Institute of Science, Bangalore and General Electric phoenix v|tome|xs at GE, Pune. Alcohol‐preserved bush frogs were carefully and tightly wrapped with bubble wrap. This package was fitted inside a jar, along with some alcohol‐soaked sponges to avoid desiccation. All specimens were scanned with the voxel size, current, and voltage adjusted according to their size (Table S1). The 3D volume files were later processed with VG Studio Max v.3.2 (Volume Graphics, Heidelberg, Germany). The skull was segmented with VG Studio Max's segmentation tools and transported as a high definition. *ply* file for use in subsequent analysis.

### 2.3 Analysis

We performed all morphometric analysis using the *geomorph* package (version 3.3.2; Adams & Otárola‐Castillo, 2013) in R (version 4.0.3) unless indicated otherwise. We digitized 36 homologous three‐dimensional landmarks on the mesh files of bush frog skulls (Figure S1). These landmarks correspond to already published landmark schema and are known to adequately capture the external skull shape (Paluh et al., 2020). The raw landmark configurations were subjected to generalized procrustes analysis (GPA). GPA superimposes the landmark configuration of all specimens using least square processes and removes size and orientation from the shape data (Adams et al., 2004). The resulting procrustes shape variables were used to perform principal component analysis (PCA).

To determine whether the mean skull shape differs between bamboo and non‐bamboo bush frogs, and to evaluate the extent to which differences in the skull shape are associated with size variation from an evolutionary perspective (evolutionary allometry), we used phylogenetic generalized least‐squares (PGLS) analysis with 1000 random residual permutations (Adams & Collyer, 2018). We used log of centroid size as a measure of cranial size. It is calculated as the square root of the sum of squared distances between each landmark point and the centroid (the mean coordinates) of all the landmark points in a shape configuration (Dryden & Mardia, 1998).

We tested the morphological disparity between the bamboo and non‐bamboo bush frogs using the *dispRity* package (version 1.7.0; Guillerme, 2018). To overcome the biases on the disparity measurement due to the sample size, we bootstrapped the data. We used nonparametric Wilcoxon test to determine the differences in morphological disparity between these two groups. To determine the rates of skull shape evolution for bamboo and non‐bamboo bush frogs and to test whether there is a rate shift in skull shape diversification associated with the Ochlandrae clade, we employed recently developed phylogenetic comparative analysis called RRphylo (Castiglione et al., 2018) using the R package *RRphylo* (version 2.7.0). This analysis uses a phylogenetic ridge regression method (see here for details, Kratsch & McHardy, 2014). It calculates the morphological evolutionary rate for each branch of the phylogeny and is intended to detect rate shifts. For the trait dataset, we used all the PCs (principal component scores) derived from landmark data, based on micro‐CT 3D skull scans from all 55 species. For the phylogenetic tree, we used a published ultrametric tree (Vijayakumar et al., 2016). We looked for rate shifts across the phylogenetic tree by screening the tree automatically for clades with significantly higher or lower rates than the rest of the tree. We further evaluated the robustness of our results generated from the above method by randomly switching the phylogenetic position of the species, eliminating a number of tips equal to 25% of the tree's size, and generating alternative phylogenies. Later, we calculated the significant shifts in the evolutionary rate for each alternative phylogeny and validated the outcomes with the original phylogeny. This procedure was iterated 100 times and the percentage of significant results was returned for each shift observed.

## 3 RESULTS

**FIGURE 1:**
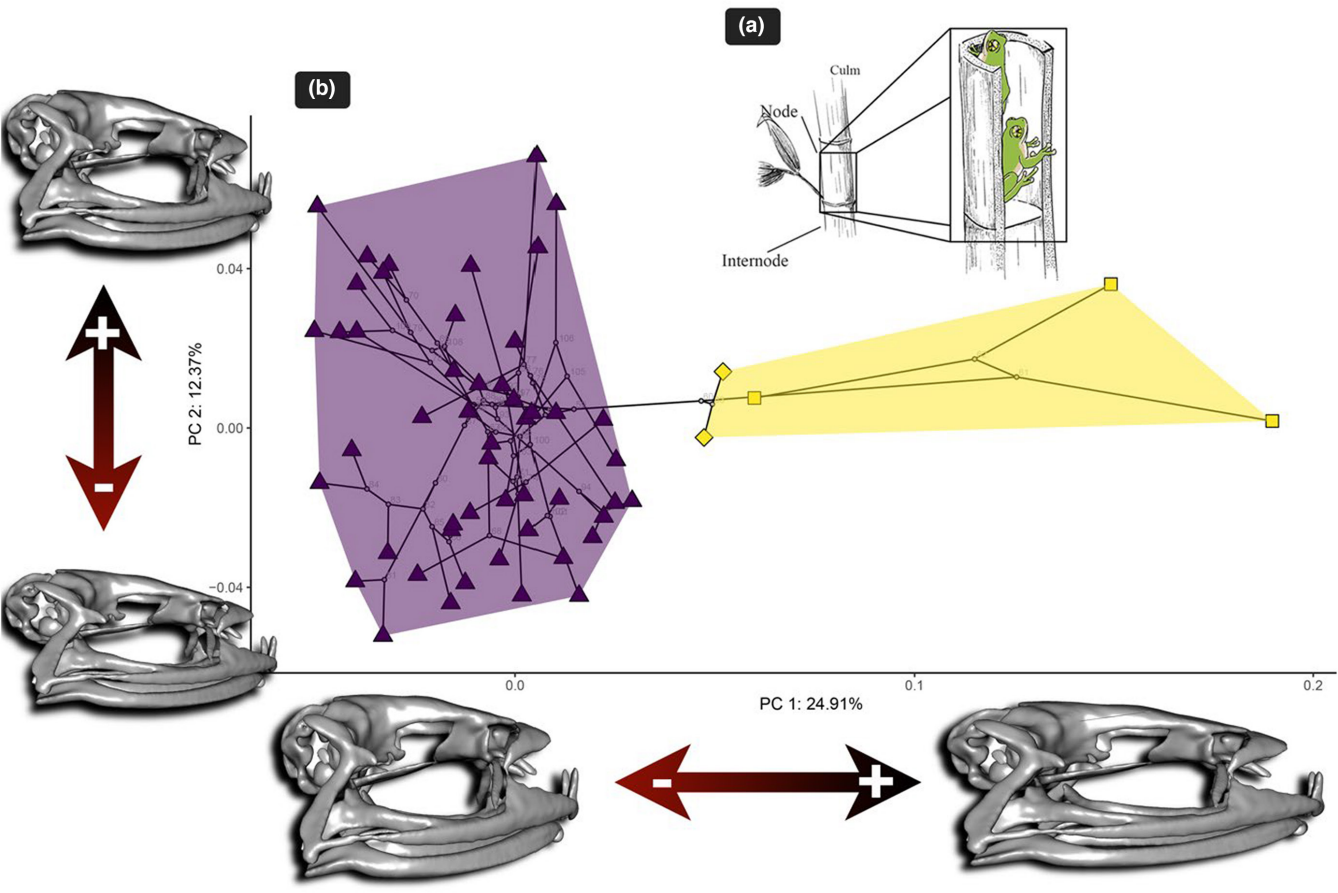
(a) The general morphology of bamboos and their utilization by bamboo‐dwelling bush frogs. (b) The phylomorphospace of the first two principal components (PCs) obtained from the phylogenetic PCA analysis of bush frog skull shape showing the clear discrimination of bamboo (enclosed in the yellow convex hull) and non‐bamboo (enclosed in the magenta convex hull) bush frogs. It also shows the two lineages of bamboo frogs (square symbol—lineage A: diamond symbol—lineage B). To visualize the shape change at both the positive and negative extremes of each axis, warped 3D meshes from an average‐shape mesh are used.

In the PCA phylomorphospace, those species inhabiting the bamboo occupied a region of shape space distinct from all other *Raorchestes* species inhabiting non‐bamboo habitats (Figure 1b). The PC1 axis explained 24.91% of overall shape variation, while the PC2 axis explained 12.37% of overall shape variation.

Along the PC1 axis, the overall skull shape was changing from long and tall (PC1 min), to board and flat (PC1 max). Some individual bones showed notable variation along the PC1 axis. The frontoparietals, which form a major portion of the skull roof, exhibited changes from a wide to a narrow structure. In the anterior region, the orbital region shifted from a narrow to a broader shape, while in the posterior region, it changed from broader to narrower. Moreover, the quadratojugal bone changed from having a short maxillary process and narrow articular surface to a long maxillary process and wide articular surface. The neopalantine changed from wider to narrower. The parasphenoid, which has two posterior laterally‐oriented processes, and an anterior cultriform process, showed variation in the shape of the cultriform process, being more lanceolate toward PC1 max, with shorter lateral processes. A variation in pterygoids could be observed in their anterior rami. They formed a straight articulation with the maxillae along PC1 min, while they were slightly curved along PC1 max. The squamosal bone has three rami: the zygomatic, otic, and ventral. Notably, the zygomatic ramus increased in length toward PC1 max, while the otic ramus underwent a change from wider to narrower, with a slight inward curve.

We detected considerable morphological changes along the PC2 axis as well. The overall skull shape was changing from flat to tall along PC2 min to max. The jaw joint was anteriorly shifted toward PC2 max. The frontoparietals exhibited variation along the PC2 axis, transitioning from a narrow and flat appearance at PC2 min to a wider and dome‐shaped structure toward PC2 max. The orbital region shifted from a round shape to an oval shape along this axis. The neopalatine bone displayed prominent changes, with a transformation from a long and expanded form at PC2 min, to a shorter and thinner form at PC2 max. Furthermore, the posterior process of the parasphenoid changed from thin and shorter to thick and elongated structure, while the anterior process was slightly acuminate (PC2 max) as compared to PC2 min. In the case of the anterior ramus of the pterygoids, subtle variations were observed along the PC2 axis, the ramus being slightly curved at PC2 min and relatively straight at PC2 max.

**FIGURE 2:**
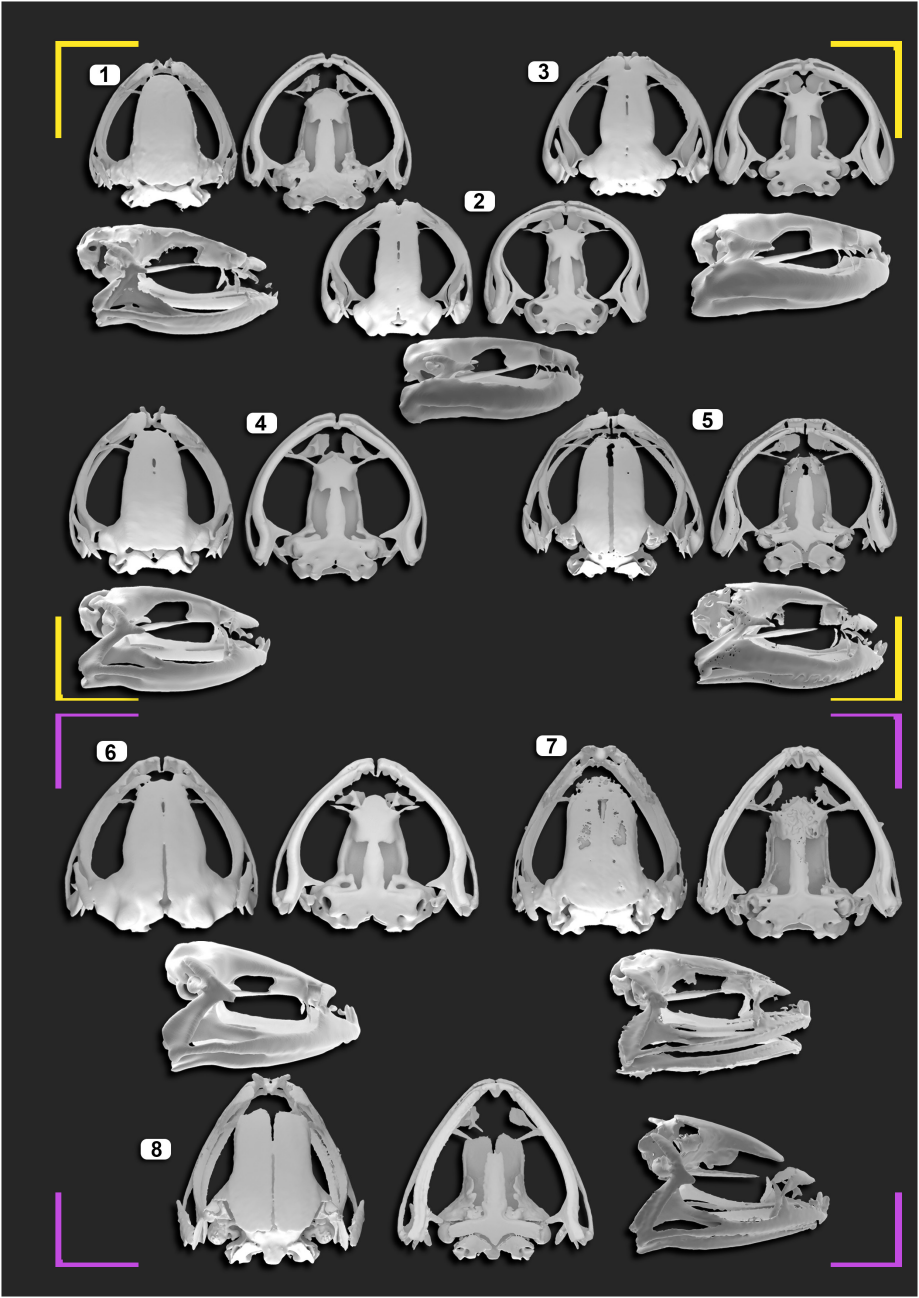
Exemplary bush frog skulls from bamboo (yellow frame) and non‐bamboo clades (magenta frame). (1) *Raorchestes uthamani* (CESF483). (2) *R. ochlandrae* (CESF2185), (3) *R. chalazodes* (CESF1198), (4) *R. manohari* (CESF2121), (5) *R. flaviocularis* (CESF1253), (6) *R. resplendens* (CESF1325), (7) *R. chotta* (CESF1057), (8) *R. archeos* (CESF 1190).

In summary, the non‐bamboo bush frogs have a long and tall skull, which is broad posteriorly and narrows anteriorly. It is characterized by a large orbital region and wide frontoparietals. Maxilla articulates with the premaxilla anteriorly, and the quadratojugal posteriorly; thus, completing the maxillary arcade. The narrow processes of paired nasals extend lateroventrally to form the anterior boundary of large orbitals and articulate with the anterior process of the maxilla (Figures 1 and 2). In the suspensorium, anterior ramus of pterygoid forms a straight articulation with maxilla. The zygomatic ramus of squamosal bone is short and otic ramus is narrow. The jaw joints are anteriorly shifted. The anterior process of parasphenoid is lanceolate with longer lateral processes. The quadratojugal bones have a short maxillary process and narrow articular surface.

The reed bamboo bush frogs deviated from the above general skull shape by an overall broader (dorsal view), and flattened (lateral view) skull. The orbital region was broader anteriorly with narrower frontoparietals. The anterior process of parasphenoid is more lanceolate with shorter lateral processes. The quadratojugal bones have long maxillary process and wide articular surface. The skull has posteriorly shifted jaw joints, with longer zygomatic ramus of squamosal and wider otic ramus. The neopalantine was narrow. The anterior ramus of pterygoid forms a curved articulation with maxilla as compared to non‐bamboo inhabitants (Figures 1 and 2).

The PGLS analysis employed to assess the impact of cranial size and habitat on cranial shape revealed a non‐significant relationship of cranial shape with habitat (*R* ^2^ = .019, *Z* = 0.425, *p* = .365), and a small but significant association with cranial size (*R* ^2^ = .042 *Z* = 1.928 *p* = .021; Table 1).

**TABLE 1:** Evaluating the effect of habitat (bamboo and non‐bamboo) and size (evolutionary allometry) on the skull shape of bush frogs using phylogenetic generalized least‐squares (PGLS) analysis (shape ~log (size) × habitat).

	df	SS	*R* ^2^	*F*	*Z*	*p*
Log (Size)	1	0.003	.042	2.320	1.928	**.021**
Habitat	1	0.002	.019	1.060	0.425	.365
Log (Size): habitat	1	0.003	.031	1.776	1.038	.154
Residuals	53	0.079	.908			
Total	54	0.086				

*Note*: Random residual permutations (1000) were used to estimate statistical significance (Bolded *p* shows significance; *p* < .05).

Abbreviations: df, degrees of freedom; PGLS, phylogenetic generalized least‐squares; SS, sum of squares.

**FIGURE 3:**
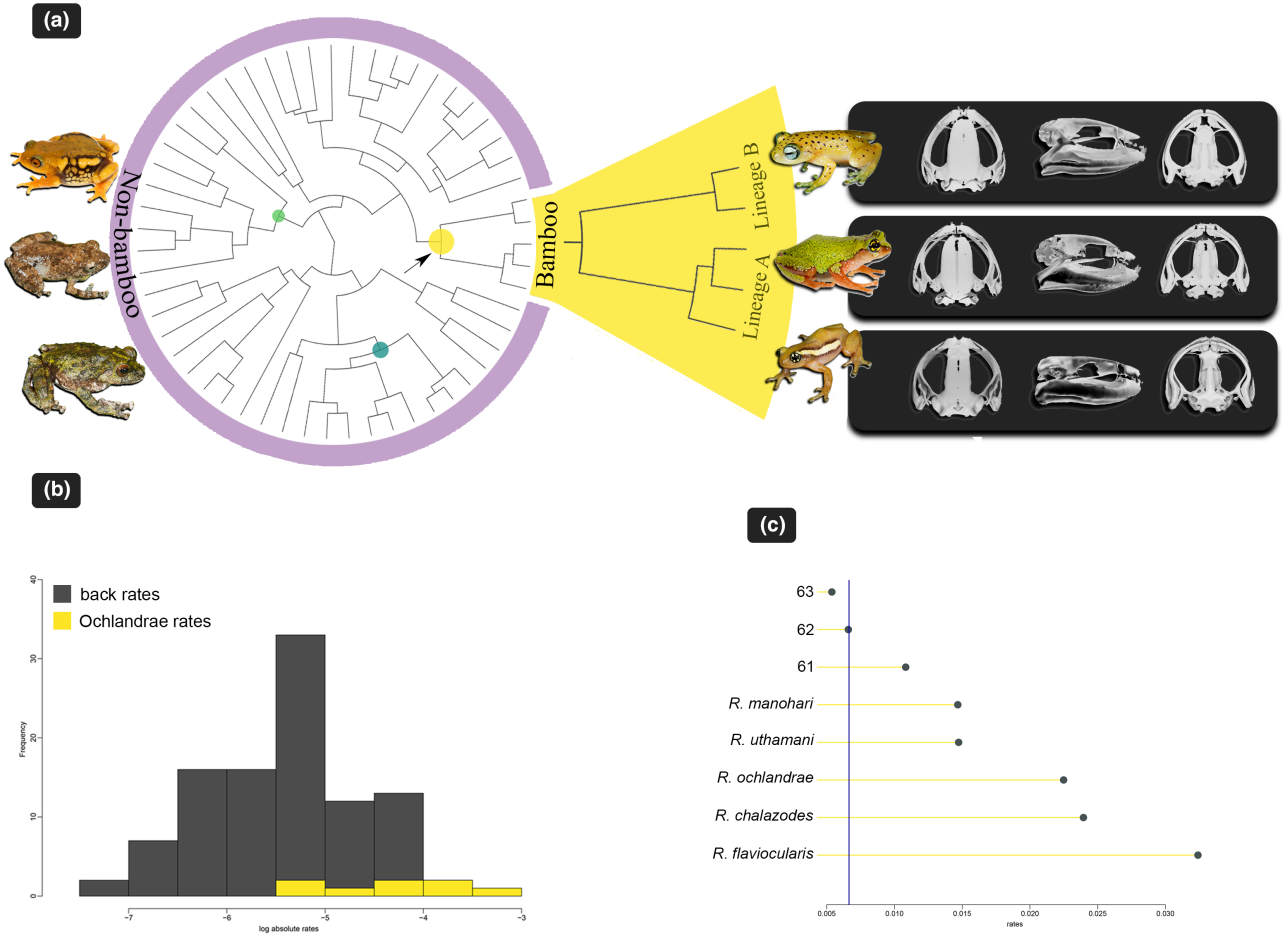
(a) Phylogenetic tree showing the rates shift computed according to phylogenetic Ridge Regression analysis. The colored dots are scaled according to the rate value (low rate = light green, to high rates = yellow). It also illustrates the two lineages (lineage A and B) within the Ochlandrae clade with example species and their skull (from the top (clockwise): *Raorchestes manohari* (CESF2121), *R. flaviocularis* (CESF1253), *R. ochlandrae* (CESF2185). On the left side are examples of frogs from non‐bamboo habitats (from the top (anti‐clockwise): *R. resplendens*, *R. chotta*, *R. keirasabinae*). (b) Phylogenetic ridge regression rates (represented in absolute values) calculated for the rest of the tree branches (gray) and are contrasted to rates for the Ochlandrae clade (yellow). The right side graph shows individual branches of the Ochlandrae clade collated in increasing rate value (yellow bars) and contrasted with the average rate computed over the rest of the tree branches (blue vertical line).

**FIGURE 4:**
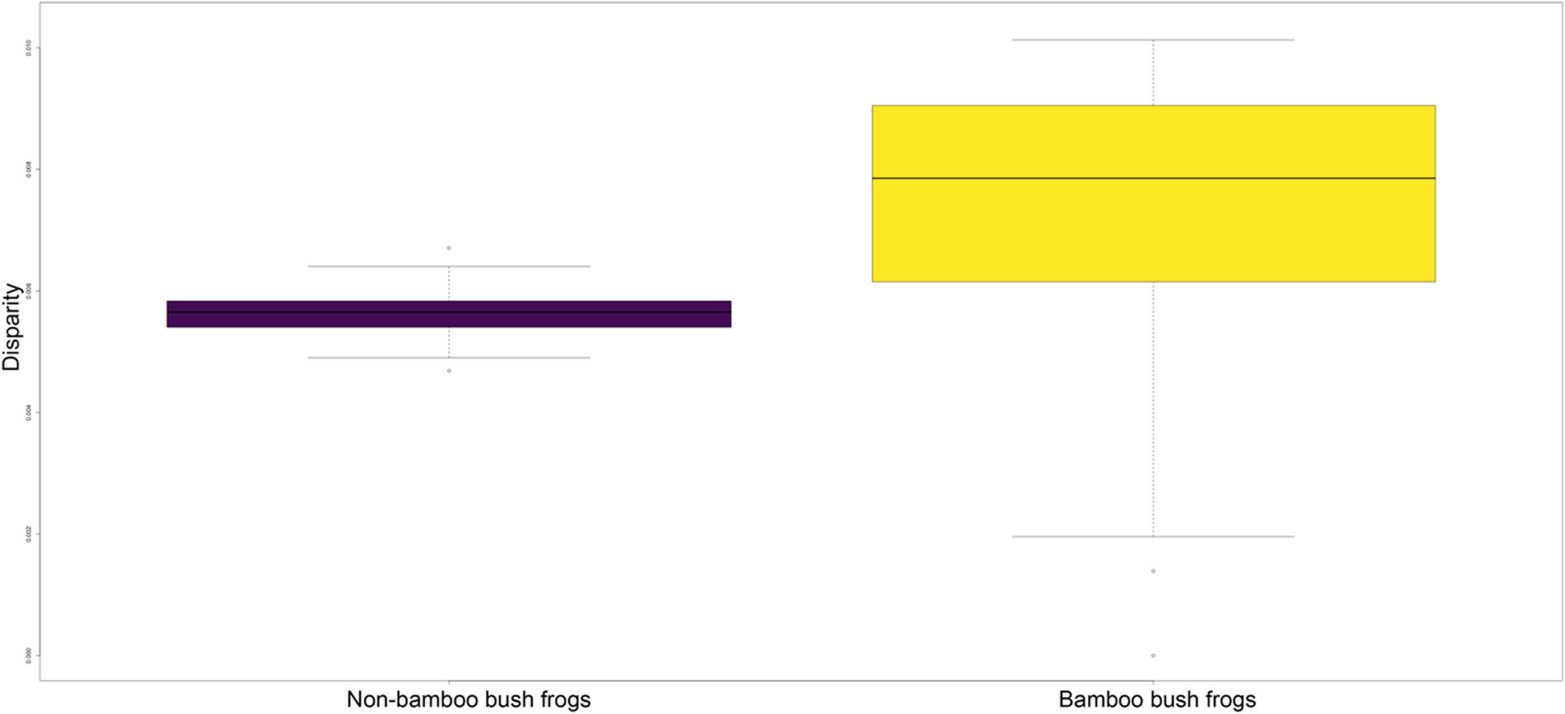
Box plot illustrating skull shape disparity (Procrustes variance) between non‐bamboo bush frogs and bamboo bush frogs (Ochlandrae). There is a significant difference in disparity (*p* < .001) between these two groups.

We subsequently applied RRphylo phylogenetic comparative analysis to model the diversification rates of bush frog skull shape and to test if there was a shift in the diversification rate of skull shape for bamboo frogs. Our results showed strong evidence for such a shift (Figure 3). The bamboo frog clade showed a remarkable increase in the skull shape evolutionary rate and shift as compared to the average rate calculated across the remaining tree branches (Figure 3). Overall, we found convincing evidence for the presence of a shift in the skull diversification rate in the monophyletic Ochlandrae frogs with 96% of the computed random trees (generated via tree swapping over 100 consecutive iterations) correctly identifying the same. We also detected a significant difference in morphological disparity between bamboo and non‐bamboo bush frogs whereas reed bamboo frogs showed higher morphological disparity (Figure 4).

## 4 DISCUSSION

Bamboo frogs breed in bamboo internodes, where they provide parental care by guarding eggs and defending them against potential predators (Seshadri et al., 2015; Seshadri & Bickford, 2018). These frogs only enter and exit the bamboo through narrow openings. Our results reveal the evolution of unique skull shapes among reed bamboo frogs. The broad, but dorsoventrally flattened skull may enable them to effectively navigate through these cavities. Remarkably, this particular skull shape has also been observed in other bamboo‐dwelling frog species, such as the casque‐headed treefrogs from the Lophyohylini tribe (Blotto et al., 2021). This intriguing pattern indicates a possible evolutionary convergence in skull shape associated with bamboo dwelling. Therefore, it would be promising to evaluate, this assumption further, for example, by testing bamboo‐dwelling frogs from Madagascar. Interestingly, bamboo bats, who roost inside bamboo internodes, display similar morphological characteristics (dorsoventrally flattened skulls) in response to their diurnal and reproductive retreats (Tu et al., 2017), thus fortifying our supposition. Apart from facilitating entry and exit from bamboo internodes, a broader and flat skull may also aid in blocking the cavities from intruders through phragmotic behavior (Seshadri & Bickford, 2018). Notable examples of such behavior have been observed in frogs of the Neotropic genera *Triprion* and *Aparasphenodon*. These frogs reside in tree holes and bromeliads, respectively, exhibiting highly derived skull shapes (Lantyer‐Silva et al., 2018; Paluh et al., 2020).

In addition to their distinct morphospace, bamboo frogs revealed an accelerated rate of morphological diversification. This remarkable shift in the evolution of the bamboo frog skull shape, compared to other bush frog species, coincides with the emergence of reed bamboos during the Miocene (Wysocki et al., 2015). Around 15 million years ago, during the mid and late Miocene, the Ochlandrae clade diversified (Vijayakumar et al., 2016). During the same period, the fossil record reveals the diversification of grasses (Bouchenak‐Khelladi et al., 2010); in particular, within the subtribe Melocanninae (Bouchenak‐Khelladi et al., 2010; Ruiz‐Sanchez & Sosa, 2015). This may have provided a new niche for mating, nesting, and offspring development for Ochlandrae frogs. Breeding habitats for frogs may be a limited resource, e.g., due to high competition and predation (Tonini et al., 2020). Consequently, the transition to a new breeding habitat represents a potential ecological opportunity leading to adaptive radiation (Tonini et al., 2020). This can be seen in certain neotropical frog species (e.g., poison frogs) whose increasing specialization toward phytotelma‐breeding has resulted in rapid diversification (Tonini et al., 2020). Therefore, it is plausible that the skull characteristics of Ochlandrae frogs have evolved in response to the use of reed bamboos as a breeding habitat, somewhere during the mid and late Miocene.

This deep‐time adaptive diversification is also supported by our phylogenetic generalized least‐squares analysis. When considering phylogenetic relationships, we observed no significant association between skull shape and habitat which is apparent in the phylomorphospace. This indicates that morphological variation in the skull shape evolved early in the evolutionary history of the Ochlandrae clade with expansion into a distinct morphospace.

Evolutionary allometry plays a significant role in shaping the cranial morphology of bush frogs. In general, they are small to medium‐sized frogs, male size ranging between 17 and 29 mm. Only a few species reach sizes of about 40 mm. The bamboo frogs range from 17 to 29 mm. Our analysis has indicated that among the smaller members of the genus, the skull shape is broader and dorsoventrally flattened, whereas in larger individuals, the skull is long and tall. This is a contrasting pattern to the suggested amphibian cranial evolutionary allometry, where large‐sized amphibians show wider skulls (Bardua et al., 2021). However, it is been known in other animal groups such as in sthenurines (short‐faced kangaroos) that adaptation to specialized ecological niches has prompted departures from the general “Cranial Evolutionary Allometry” rule (large size‐long face) for mammals (Cardini et al., 2015). Consequently, we postulate that the “small size‐wide skull” phenomenon, observed in bamboo frogs, is a direct consequence of their adaptive strategy, tailored to navigating their bodies through small cavities of bamboo internodes.

Contrary to our initial expectation based on shared ancestry and similar habitats, reed bamboo frogs showed higher, not lower, morphological disparity compared to non‐bamboo bush frogs. The clade consists of two lineages: lineage A comprises three species (*R. chalazodes*, *R. flaviocularis*, and *R. ochlandrae*), and lineage B comprises the two remaining ones, *R. manohari* and *R. uthamani*. The two lineages split early (Mid‐Miocene, Vijayakumar et al., 2016) and this deep divergence is observed in the phylomorphospace. The two species in lineage B, *R. manohari* and *R. uthamani*, are allopatric sisters with narrow ranges, and as expected from our deep‐time adaptive diversification hypothesis, they show conservatism in skull shape. However, the species in lineage A show a complex pattern of greater separation in morphospace. Within lineage A, *R. ochlandrae* is distributed widely and occurs in low to mid elevations, whereas the other two species have narrow distributions and are allopatric sisters in high elevations (Vijayakumar et al., 2016). A further parameter that could potentially influence the cranial morphology of reed bamboo frogs pertains to the distinct characteristics of various bamboo species. There are 10 endemic reed bamboo species, *Ochlandra* spp., within the range of the bamboo frogs (Bouchenak‐Khelladi et al., 2010). Further investigation is required to explore the intricate relationships between bamboo and frogs, as well as the specific characteristics of bamboo plants. This includes factors such as typical dimensions of cavities and internode measurements that might affect morphological diversification of the bamboo frogs.

Apart from the bamboo habitat, additional factors such as diet have the potential to exert an influence on the skull shape evolution of bamboo frogs. Although information regarding their diet remains limited, it is known that they primarily consume small invertebrates. However, there is evidence of *R. chalazodes* (lineage A) feeding on the large semi‐slug, *Satiella dekkanensis* (Seshadri, 2020). It has been postulated that the consumption of larger prey, particularly preceding the breeding phase, could confer advantages to adult males during the ensuing phase of parental care within bamboo internodes, where sustenance might be scarce or absent. Whether similar dietary patterns are occasionally observed in other Ochlandrae species, or if this behavior is specific to *R. chalazodes*, remains an unresolved question.

We acknowledge that a limitation of our study group is the single evolutionary transition of bush frogs breeding outside of bamboo to a lineage breeding within bamboo internodes. These singular events have been the perennial foci in comprehending the results of phylogenetic comparative methods and are under much debate (Addison & Fitzjohn, 2015; Uyeda et al., 2018). Nonetheless, our study offers novel insights into the skull‐shape diversification of bamboo frogs based on their tight association of life history with *Ochlandra* bamboo. Furthermore, we believe that our study system of a novel evolutionary innovation offering notable adaptive advantages, i.e., living and breeding in an exclusive new type of habitat not accessible to others, has a unique potential for replication. Considering the presumed benefits of the skull characteristics of bamboo frogs, we hypothesize that other, unrelated frog clades inhabiting bamboos, may have evolved a similar morphology. This could for instance be further tested with a variety of specialized microhylid frogs from Madagascar.

## 5 CONCLUSIONS

In summary, our findings highlight the potential influence of evolutionary history, ecological opportunity, and reproductive behavior on the cranial morphology of bush frogs inhabiting reed bamboo internodes. Our research has unraveled that the plant‐frog relationship is a result of deep‐time ecological opportunity dating back to the Miocene, and triggered specific skull shape, deviating from other members of the frog genus *Raorchestes*.

## AUTHOR CONTRIBUTIONS

**Kalpana Das:** Conceptualization (equal); data curation (lead); formal analysis (lead); funding acquisition (equal); investigation (equal); methodology (equal); project administration (equal); resources (equal); software (equal); supervision (equal); validation (equal); visualization (lead); writing – original draft (lead); writing – review and editing (equal). **Mark‐Oliver Rödel:** Conceptualization (equal); data curation (supporting); funding acquisition (equal); investigation (equal); methodology (supporting); project administration (equal); resources (equal); software (equal); supervision (equal); visualization (supporting); writing – review and editing (equal). **Edward Stanley:** Data curation (equal); methodology (equal); software (equal). **Achyuthan N. Srikanthan:** Data curation (equal); software (supporting). **Kartik Shanker:** Conceptualization (equal); funding acquisition (equal); writing – review and editing (equal). **Seenapuram Palaniswamy Vijayakumar:** Conceptualization (equal); data curation (equal); formal analysis (equal); funding acquisition (equal); investigation (equal); methodology (equal); project administration (equal); resources (equal); software (equal); supervision (equal); validation (equal); writing – review and editing (equal).

## Supporting information


Appendix S1

Video S1

## Data Availability

CT scan data of some of our specimens are available on Morphosource.org. Surface meshes and CT scan data of others are available on the MfN data repository. Phylogenetic tree data are available from the previous publication (Vijayakumar et al., 2016) under https://doi.org/10.5061/dryad.8r3v0. R code and associated data analysis files are available on https://github.com/Kp‐Das/Bamboo‐frogs.
