# Supplementary material for: Reed bamboos drive skull shape evolution in bush frogs of the Western Ghats, Peninsular India
Source: Ecol Evol. 2023 Sep 5;13(9):e10493. doi: 10.1002/ece3.10493 (PMC10480065; doi:10.1002/ece3.10493)
Supplement: Supplementary file 1 — Appendix S1 [file ECE3-13-e10493-s001.docx]

**Supplementary Information**

Reed Bamboos drive skull shape evolution in bush frogs of the Western Ghats, Peninsular India

This PDF file contains the following information:

**Table S1-S2**

**Figure S1**

**Table S1** Specimens used for obtaining CT-scans including the repository code (CESF- Centre for Ecological Sciences Frogs), scan availability, associated clades and broad habitat associations.

**Fig. S1** Landmarks selected on the bush frog skull in different perspectives: a) Dorsal b) Ventral c) Anterior d) Lateral e) Posterior using the R function *digit.fixed* in *geomorph* package (version 3.3.2) . The corresponding landmark definitions (matched with landmark numbers here) are provided in the Table S2. The example skull shown here is of *Raorchestes ochlandrae* (CESF2185).


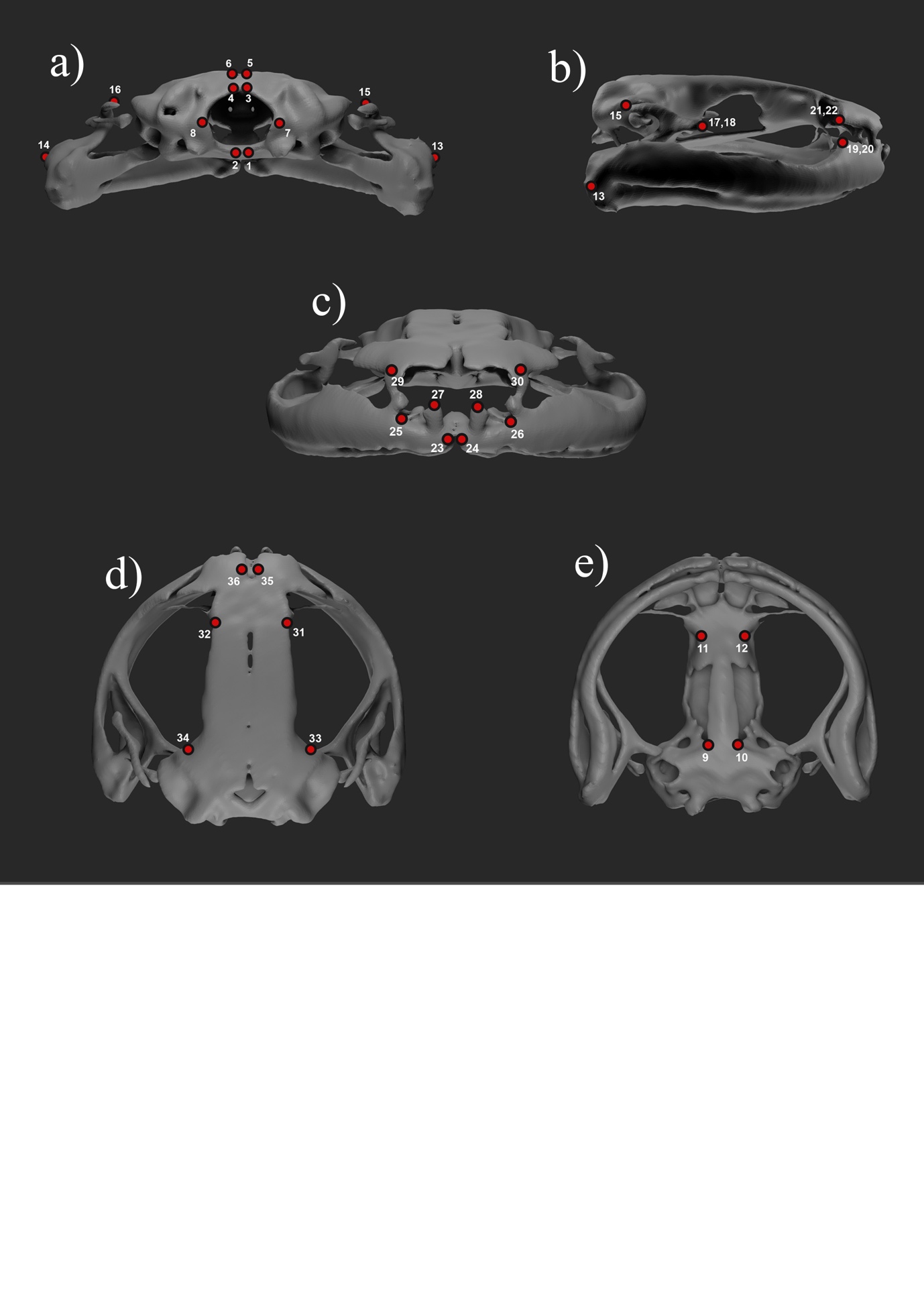


**Table S2** Landmark numbers with their corresponding position and bone.

| **Landmark no.** | **Landmark description** | **Bones** |
| --- | --- | --- |
| 1,2,3,4 | margins of foramen magnum | occipital bone |
| 5,6 | posterior region of skull roof | skull roof |
| 7,8 | opposite ends of occipital condyle | occipital condyle |
| 9,10 | posterior end of parasphenoid | parasphenoid |
| 11,12 | anterior end of parasphenoid | parasphenoid |
| 13,14 | jaw joint, left and right | quadrate |
| 15,16 | tip of posterior (otic) ramus of squamosal, left and right | squamosal |
| 17,18 | tip of anterior(zygomatic) ramus of squamosal, left and right | squamosal |
| 19,20 | preorbital process of the maxilla, left and right | maxilla |
| 21,22 | maxillary process of the nasal, left and right | nasal |
| 23,24 | rostral end of premaxilla, left and right | premaxilla |
| 25,26 | rostral end of maxilla/ caudal end of premaxilla, left and right | maxilla/premaxilla |
| 27,28 | tip of alary processes of premaxilla, left and right | premaxilla |
| 29,30 | anterior extent of the nasals | nasal |
| 31,32 | anterolateral extent of the sphenethmoid | sphenethmoid |
| 33,34 | posterolateral extent of the frontoparietal | frontoparietal |
| 35,36 | anterior extent of the sphenethmoid | sphenethmoid |
